# Helping or punishing strangers: neural correlates of altruistic decisions as third-party and of its relation to empathic concern

**DOI:** 10.3389/fnbeh.2015.00024

**Published:** 2015-02-18

**Authors:** Yang Hu, Sabrina Strang, Bernd Weber

**Affiliations:** ^1^Center for Economics and Neuroscience, University of BonnBonn, Germany; ^2^Department of Psychology, University of LübeckLübeck, Germany; ^3^Department of Epileptology, University Hospital BonnBonn, Germany

**Keywords:** third-party help, third-party punishment, empathic concern, reward, striatum

## Abstract

Social norms are a cornerstone of human society. When social norms are violated (e.g., fairness) people can either help the victim or punish the violator in order to restore justice. Recent research has shown that empathic concern influences this decision to help or punish. Using functional magnetic resonance imaging (fMRI) we investigated the neural underpinnings of third-party help and punishment and the involvement of empathic concern. Participants saw a person violating a social norm, i.e., proposing unfair offers in a dictator game, at the expense of another person. The participants could then decide to either punish the violator or help the victim. Our results revealed that both third-party helping as well as third-party punishing activated the bilateral striatum, a region strongly related with reward processing, indicating that both altruistic decisions share a common neuronal basis. In addition, also different networks were involved in the two processes compared with control conditions; bilateral striatum and the right lateral prefrontal cortex (lPFC) during helping and bilateral striatum as well as left lPFC and ventral medial prefrontal cortex (vmPFC) during punishment. Further we found that individual differences in empathic concern influenced whether people prefer to help or to punish. People with high empathic concern helped more frequently, were faster in their decision and showed higher activation in frontoparietal regions during helping compared with punishing. Our findings provide insights into the neuronal basis of human altruistic behavior and social norm enforcement mechanism.

## Introduction

Humans have an intriguingly complex social norm system, which is unique in the animal kingdom and essential for the functioning of human society (Fehr and Rockenbach, 2004). Self-interests are often in conflict with these social norms. When allocating resources our self-interests might lead us to favor an unequal distribution at the expense of others, violating fairness or equal distribution norms. When observing another person violating a social norm, e.g., treating another person unfair, we have at least two options of how to react to this norm violation, namely to either punish the offender, or to help (compensate) the victim. Punishing the offender is referred to as retributive justice (Hogan and Emler, 1981) and helping the victim is referred to as compensatory justice (Darley and Pittman, 2003). Usually people have to choose whom they want to focus on (i.e., the offender or the victim) and then decide whether they want the offender to pay for what he or she did, or whether they want to restore the harm done to the victim (Schroeder et al., 2003). It was shown that people's first reaction to norm violations of high severity is to punish the offender. However, people have a desire to help the victim after norm violations of low severity or when asked to focus on the victim (Gromet and Darley, 2009). Furthermore, victims themselves attach importance to being helped or compensated (Umbreit, 1998). Thus, both punishing the offender as well as helping the victim are conceivable reactions to norm violations and might help to restore social equity.

Helping a victim as well as punishing a norm violator as a third-party (outside observer) can be regarded as altruistic acts. Both cost people at least time and effort but provide no direct benefits. Nevertheless, people show altruistic helping (Leliveld et al., 2012) as well as altruistic punishment (Fehr and Gächter, 2002; Fehr and Fischbacher, 2004). Both behaviors reduce inequality between offender and victim. Recent neuroimaging studies suggest that altruistic behavior is intrinsically rewarding as it was found to be correlated with activity in the striatum, an area known to be involved in reward processing (Haber and Knutson, 2010). Specifically, the ventral striatum was shown to be activated when people invest their own money to reduce their teammates' physical pain (Hein et al., 2010) and when helping an African orphan (Genevsky et al., 2013). Although the first-party is not explicitly mentioned in these studies, helping in this context can be regarded as a form of third-party helping. Participants were not involved in the unfair situation themselves (they were neither victims nor violators) but helped another victim. De Quervain and colleagues found that the striatum was also involved in second-party punishment, namely when participants punished the untrustworthy opponent in a trust game paradigm (De Quervain et al., 2004). In this case the participant was the victim of unfair behavior. So far, there are only two studies on the neural correlates of third-party punishment (Buckholtz et al., 2008; Strobel et al., 2011). In the study by Buckholtz and colleagues, participants were asked to rate the appropriate punishment for crimes they were not involved in. However, decisions in this study were not incentivized and therefore not costly for participants. In another study by Strobel and colleagues, a modified paradigm of dictator game was adopted in which participants played the role of either the recipient (i.e., second-party) or the observer (i.e., third-party) and they could punish the dictator with their own money. They found that both second-/third-party punishment (vs. no punishment) elicited stronger activation in ventral striatum. Thus, up to now neuroimaging studies show that second-party punishment and third-party help involve similar neuronal processes, namely activity in reward areas. Reward might be an underlying mechanism for both third-party help and punishment decisions, thus both might involve activity in the striatum.

Despite some similarities, behavioral studies suggest that third-party altruistic punishment and help seem to be driven by different motives. On the one hand people feel sympathy/empathy with the victim triggering a desire to restore the person (Gromet and Darley, 2009). On the other hand norm violations induce strong negative affect which lead people to punish the offender (Fehr and Gächter, 2002; Egas and Riedl, 2008). One additional motive of punishment is deterrence; punishment has the additional function to prevent offenders from future norm violations (Carlsmith et al., 2002). Taken together, behavioral studies suggest that third-party help and punishment are differently motivated and might therefore involve different processes. Intriguingly, people differ in their responses when asked to choose between punishing the offender and helping the victim of a norm violation. A recent behavioral study found that when witnessing an unfair case of monetary allocation, people as third-parties with low empathic concern preferred punishment, whereas those high in empathic concern preferred helping (Leliveld et al., 2012). This indicates that empathic concern plays an important role in influencing people's choice either to help or to punish. Empathic concern is defined as an other-oriented altruistic motivation congruent with the perceived welfare of another person; namely a feeling of concern for other people who are in need or suffer from an unfortunate case (Coke et al., 1978; Batson et al., 1988). More crucially, previous studies have shown that empathic concern is a reliable indicator for helping behavior (Coke et al., 1978; Batson et al., 1983; Eisenberg and Miller, 1987; Batson et al., 1988). As a stable disposition variable, empathic concern was measured by one subscale of the Interpersonal Reactivity Index (IRI_EC Davis, 1983). The IRI_EC was also used in previous neuroimaging studies to investigate correlations between empathic concern and empathic neural responses, however the results are inconclusive. One of the main reasons is that different approaches were used in those studies to assess the neural correlates of empathy, which makes it difficult to compare the results (Singer et al., 2004; Lamm et al., 2007; Decety, 2011). For example, Singer et al. (2004) adopted a cue-based paradigm, in which participants' empathy was elicited by abstract visual information about their partner's affective state. They found stronger positive relation between IRI_EC scores and empathic neural activities in anterior cingulate cortex and left anterior insula. In the study by Lamm et al. (2007), a picture-based paradigm was used, in which participants' empathy was elicited by viewing other's body parts in painful situations (e.g., the painful needle injection on someone's hand). However, no correlation was found between IRI_EC and empathic neural activities in those regions.

Although third-party help and punishment have been extensively investigated in behavioral studies, the neuronal basis of third-party help and punishment has not been examined simultaneously in one study using the same paradigm so far, allowing for a direct comparison. Furthermore, the association between empathic concern and brain responses to third-party help or punishment is still unclear. Adapting the paradigm of Leliveld et al. (2012) we investigated the neural correlates of third-party help and punishment simultaneously in one study by using fMRI. Our aim was to examine the neural processes underlying third-party help and punishment and their relation to individual differences in empathic concern. Based on previous neuroimaging research we hypothesize that both third-party help as well as punishing activates the striatum (De Quervain et al., 2004; Genevsky et al., 2013). However, since behavioral studies showed that the motives to punish and help are different we predict that help and punishment elicit activity in separate brain regions connected to the striatum. Furthermore, we assume that individual differences in empathic concern correlate with both the frequency of help decisions and brain activity related to help (vs. punishment). Given that previous studies do not report any consistent results about possible target regions for the connectivity analyses, we refrain to make strong predictions but rather choose to present exploratory results.

## Materials and methods

### Participants

Thirty-six German participants (12 males; mean age = 22.72 ± 2.85) were tested in the fMRI experiment. All participants reported no history of psychiatric or neurological disorders. They were recruited via the Online Recruitment System for Economic Experiments (ORSEE). Written consent was given by all participants according to the Declaration of Helsinki (BMJ 1991; 302: 1194) and the study was approved by the ethics committee of the University of Bonn. Additional 84 participants (30 males; mean age = 23.58 ± 6.13) were recruited for the behavioral experiment from the same subject pool as used for the fMRI experiment.

### Stimuli and design

The experiment consisted of two parts: a behavioral and an fMRI part. Participants of the behavioral part were asked to play a Dictator Game. During ten rounds half of them played the role of the proposer (i.e., first-party) and the other half the role of the recipient (i.e., second-party). We used a perfect stranger matching to allocate participants for each round. The proposer received an endowment of 100 monetary units (MUs; 1MU = 20 Cents) per round and could decide how to distribute these between him-/herself and the recipient (i.e., 0, 10, 20, 30, 40, 50). Participants were informed that some of their decisions were forwarded to a third-party (i.e., the fMRI participants). In case of an unfair allocation the third-party could decide to either help the recipient by transferring MUs to increase the recipients' original MUs or to punish the proposer by investing own MUs to subtract the proposers' original MUs. Participants were further asked to indicate their initials and were informed that these were forwarded to the third-parties. All participants of the behavioral experiment received a 4 € show-up fee at the end of the experiment. They were also informed that in addition all parties would receive payoffs depending on one randomly chosen round of the experiment. Thus, if the third-party decided to either help the recipient or punish the proposer this decision was implemented accordingly. The additional payoffs (*M* = 10.05 €, SD = 7.26 €) for participants of the behavioral experiment were paid four weeks later. The behavioral part of experiment was conducted in Bonn EconLab via Z-tree (Fischbacher, 2007).

In total, 420 decisions were made by the proposers, including 63 decisions of 50/50 offer, 43 offers of 60/40 offer, 33 decisions of 70/30 offer, 57 decisions of 80/20 offer, 82 decisions of 90/10 offer and 142 offers for 100/0. Given the goal of our study and the fMRI design, we focused on the unfair offers (i.e., 60/40, 70/30, 80/20, 90/10, 100/0) and selected 160 offers to present those in the fMRI study. Among them, 120 offers were presented in the decision condition and 40 in the control condition. Each offer (i.e., 60/40, 70/30, 80/20, 90/10, 100/0) occurred 24 times in the decision condition and 4 times in the control condition.

### fMRI procedure

Participants were informed about the behavioral experiment and that they would see a set of allocations made during this experiment. They were further told that they could influence the payoff of either the first- or second-party by investing their own endowment. Importantly, both options were costly for the participant, meaning that they had to invest one MU in order to either subtract three from the proposer or to increase three to the recipient. Prior to the scanning session, participants received an instruction which included a short comprehension test to further make sure that they understood the task.

**Figure 1:**
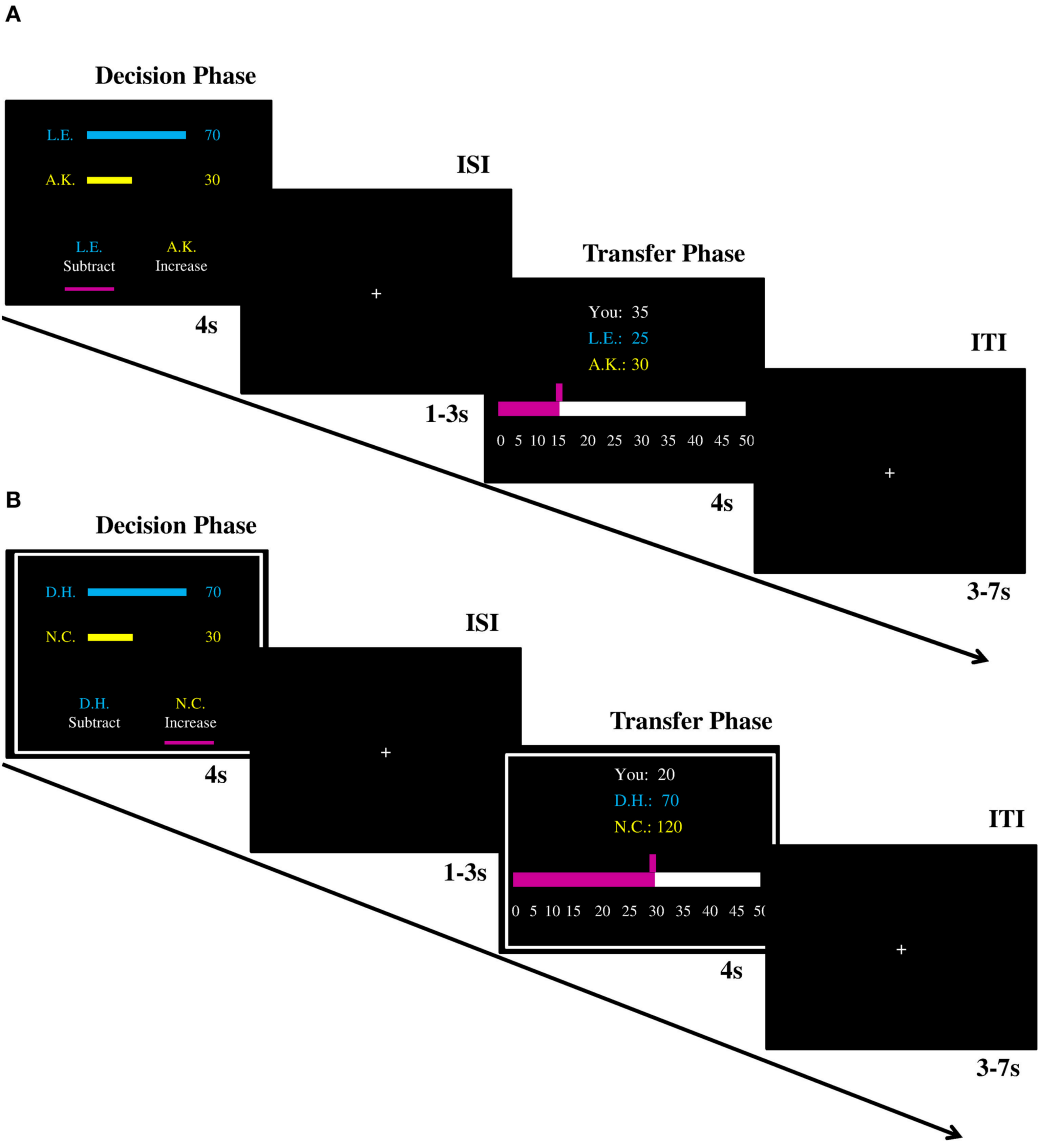
**Illustration of trial procedures (A) in the decision condition and (B) in the control condition**. ISI, inter-stimulus interval; ITI, inter-trial interval.

The scanning session consisted of two fMRI runs, which were separated by a self-paced break. In each run, there were 80 trials; 60 decision trials (12 trials per offer) and 20 control trials (4 trials per offer, half of them were in help/punish condition). In each trial, participants were endowed with 50 MU (1MU = 20 Cents). In the decision condition, participants first saw the unfair monetary allocation paired with the initials of the first- and second-party (Figure 1A). On the same screen they were asked whether they wanted to increase the recipient's payoff or to decrease the proposer's payoff. Once they made a choice, a cue appeared under the corresponding option (the decision phase). Independent of their response time the decision phase was presented for 4 s. The decision phase was followed by an inter-stimulus fixation cross (1–3 s). On the next screen participants could decide how much they want to increase or decrease the payoffs of the other players (the transfer phase; 4 s), followed by an inter-trial fixation cross (3–7 s). Participants could respond by pressing the button of response grips with the left/right index fingers in both phases of the task. In the control condition, the procedure was identical except that in both phases decisions were made by the computer instead of the participants lying in the scanner. The offers presented during these trials were still made by the participants of the behavioral experiment though. Thus, participants in the scanner did not make any decisions themselves, however, these trials were relevant for the payoffs of all parties (proposer, recipient and fMRI participant). Participants therefore had an incentive to keep track of the control condition trials. No button presses were asked of the participants in the control condition to limit the feeling of a forced choice which might lead to conflict, anger or frustration. These trials were indicated by a white frame (Figure 1B). The display of the task and response collection was performed with Presentation 14.9 (Neurobehavioral System, Albana, Canada). Participants saw the experiment via video goggles (Nordic NeuroLab, Bergen, Norway) and their responses were recorded by response grips (Nordic NeuroLab, Bergen, Norway).

It is important to highlight the following details of the paradigm and the procedure. First, the words “help” and “punish” were not used in the instructions (“increase” and “subtract” were adopted instead) to avoid demand characteristics. Second, consistent with previous literature (Fehr and Fischbacher, 2004; Leliveld et al., 2012), the cost ratio was set to 1:3, which means that 1 MU transferred from participants could either subtract 3 MU from the first-party or increase 3 MU to the second-party. Third, in the transfer phase participants could decide to invest 0 MU. Thus, every decision to invest MUs to either increase or decrease MUs of the others can be regarded as their voluntary decision. Fourth, the position of two options (i.e., “increase” and “subtract”) in the decision phase were counterbalanced across trials. The default position of the amount participants could invest in the transfer phase was randomly determined from 0 to 50. Finally, the first-party could not lose money (i.e., the minimum payoff was 0).

After scanning, participants were asked to fill in the Interpersonal Reactivity Index (IRI) scale, used for measuring trait empathy and to make judgments about the fairness of the six different offers (i.e., the offer 50/50 was also included) on a 8-point Likert scale (1 = very fair, 8 = very unfair). Finally, participants received a 10 € show-up fee and one randomly selected trial was paid to all three parties accordingly (*M* = 7.0 €, SD = 2.5 €).

### Data collection and analyses

The imaging data was collected via the 3-Tesla Siemens Trio platform at the Imaging Center of Life & Brain, University Hospital Bonn. For functional images, 37 axial slices (FOV = 192 × 192 mm^2^, matrix = 96 × 96, in-plane resolution = 2 × 2 mm^2^, thickness = 3 mm) covering the whole brain were obtained using a T2^*^-weighted echo planar imaging (EPI) sequences with blood-oxygenation-level dependent (BOLD) contrast (TR = 2500 ms, TE = 30 ms, flip angle = 90°). A high-resolution structural image for each participant was acquired using 3D MRI sequences for anatomical co-registration and normalization (TR = 1660 ms, TE = 2.75 ms, flip angle = 9°, matrix = 320 × 320, FOV = 256 × 256 mm^2^, slice thickness = 0.8 mm).

Eleven participants were excluded due to the following reason: 10 of them had insufficient number of trials in both runs (less than 5 trials) for one or both decision regressors (help decision: *n* = 1; punish decision: *n* = 7; both decisions: *n* = 2) and one participant terminated the experiment because he or she felt uncomfortable in the scanner. For the remaining 25 participants, SPM8 was used for the fMRI data analysis (Welcome Trust Department of Cognitive Neurology, London, UK). For each run of each participant, the first three volumes were discarded to allow the stabilization of BOLD signal. The following preprocessing steps were applied: EPI images were first realigned to the first volume to correct for head motions (<2.5 mm) and corrected for slice timing. Then, the anatomical image was co-registered to the mean EPI image, and segmented, generating parameters for normalization to MNI space. Using these parameters, all EPI data were projected onto MNI space with a 2 × 2 × 2 mm^3^ resolution and smoothed using an 8-mm FWHM (full width half maximum) isotropic Gaussian kernel. High-pass temporal filtering with a cut-off of 128 s was performed to remove low-frequency drifts.

For the individual-level analyses, a general linear model (GLM) focusing on the decision-phase with five onset regressors (i.e., “help,” “punish,” “help_control,” “punish_control,” “other”) convolved with the canonical hemodynamic response function (HRF) was applied. The “other” regressor included the following onsets: onsets of transfer phase and onsets of no response as well as trials in which participants transferred 0 MU in decision phase. For runs in which either “help” or “punish” condition was less than 5 trials, onsets of that condition in decision phase were also categorized into “other” condition. The six estimated head movement parameters were included in the design matrix to account for the residual effects of head motion. For the group-level analyses, a one-sample *t*-test as well as a flexible factorial model was performed to test the difference and the conjunction of the activation elicited by “help” and “punish” option. Parameter estimates (contrast values) and percent signal change of the peak voxel was extracted via MarsBar (http://marsbar.sourceforge.net).

#### Correlation analysis

To investigate how trait empathy correlates with third-party decisions at the neural level, a correlation analysis was applied to compute the relationship between the individual neural contrast of “help” vs. “punish” and individual scores of empathic concern subscale of the IRI (IRI_EC).

#### Psycho-physiological interaction (PPI) analysis

In order to test whether different networks are involved during helping and punishing respectively, we performed a PPI analysis (Friston et al., 1997; Gitelman et al., 2003). Specifically, the source masks were defined as two 8-mm spheres centered at the peak voxel of the group-level conjunction results of the two contrasts “help” vs. “help_control” and “punish” vs. “punish_control” within bilateral striatum based on AAL templates with the wfu_pickatlas tool. The seed volume of interest (VOI) for each individual was then defined as a sphere with a 6-mm-radius centered at the peak voxel from the contrast of either “help” vs. “help_control” or “punish” vs. “punish_control” within these source masks. The time series of each VOI was extracted and then deconvolved, multiplied with the psychological variable (“help” > “help_control” or “punish” > “punish_control”) and reconvolved with a hemodynamic response function to set up the PPI regressor, which followed the procedure by Gitelman et al. (2003). These three regressors (i.e., the PPI regressor, the VOI time-series, the psychological variable) were convolved with the canonical HRF and then entered into the regression model along with six head motion parameters. The individual parameter estimates image for the PPI regressor was subsequently subjected to one-sample *t*-tests. Finally, a group analysis was performed to identify the brain regions displaying increased functional connectivity with the seed VOI during either help or punishment decisions. Besides, two paired-samples *t*-tests were performed to further test the different connectivity patterns between help and punishment decisions with either left or right striatum.

For all whole-brain based analyses mentioned above, the threshold of *p* < 0.001 uncorrected at peak voxel level with the extent threshold at *k* = 50 was adopted.

## Results

### Behavioral results

Data from 25 participants were used for behavioral analyses. A paired-samples *t*-test was performed between help and punishment decisions in the decision condition on the behavioral factors *decision rate* (i.e., the ratio of help/punish decision compared in relation to all respective trials), *response time* (ms) and *transfer amount* (MU). The *transfer amount* was significantly different between help and punishment trials. Participants transferred more MUs when they punished the first-party (*M* = 16.15, SD = 6.86) than when they helped the second-party (*M* = 11.07, SD = 5.07) [95% C.I. of the difference: −8.28 to −1.89; *t*_(24)_ = 3.266, *p* = 0.003, Cohen'd = −0.664]. No significant differences were detected in the *decision rate* (help: *M* = 49.30%, SD = 27.28%; punish: *M* = 42.40%, SD = 27.90%) [95% C.I. of the difference: −15.62–29.42%; *t*_(24)_ = 0.632, *p* = 0.533, Cohen'd = 0.126] and *response times* (help: *M* = 1583.15, SD = 431.63; punish: *M* = 1611.45, SD = 402.22) [95% C.I. of the difference: −207.55–150.93; *t*_(24)_ = −0.326, *p* = 0.747, Cohen'd = −0.065] between help and punishment trials.

**Figure 2:**
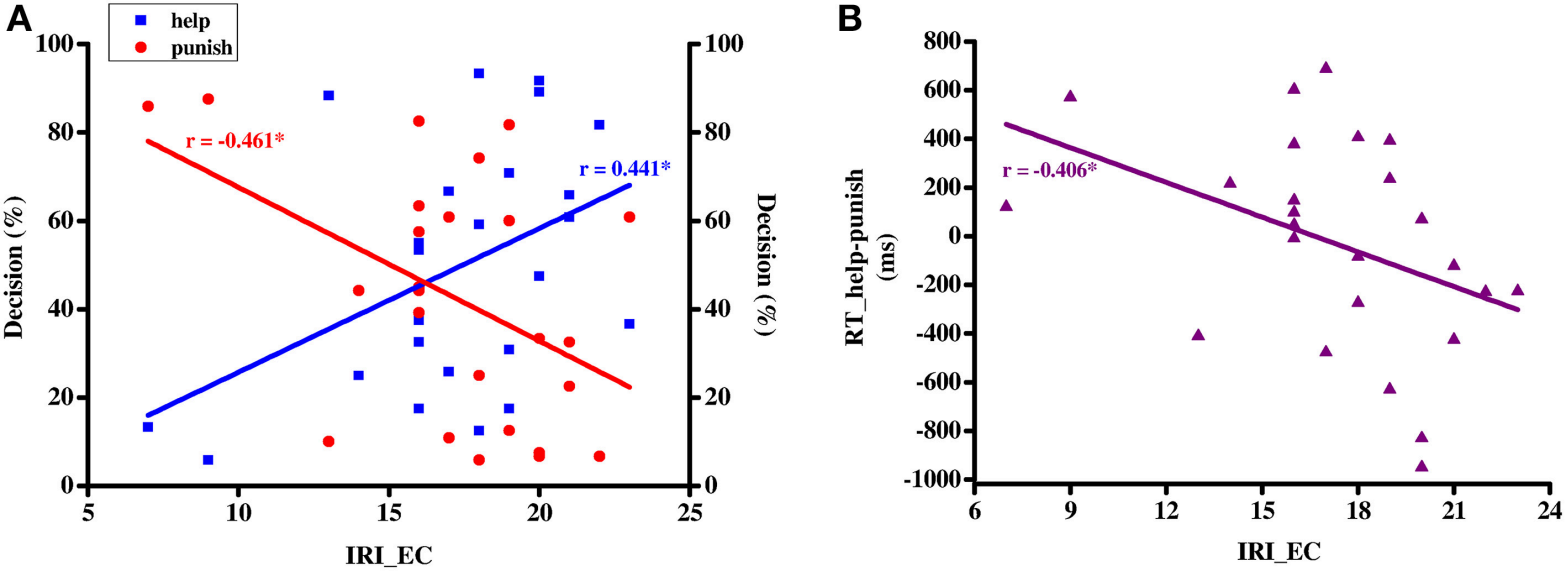
**Correlation (A) between IRI_EC scores (X-axis) and average help/punish rate (Y-axis) and (B) between IRI_EC scores (X-axis) and the difference in RT between help and punish (Y-axis)**. IRI_EC, empathic concern subscale of interpersonal reactivity index scale; RT, response time; ^*^p < 0.05.

To test whether individual differences in trait empathy correlate with the decisions to help or punish, a Pearson correlation was conducted between empathic concern subscale scores of the IRI (i.e., IRI_EC) and *decision rate* in help and punishment decisions respectively. A significant positive relationship was found between IRI_EC scores and *help rate* [95% C.I.: 0.06–0.71; *r* = 0.441, *p* = 0.027, Fisher's *Z*_r_ = 0.474], whereas a negative relationship was detected between IRI_EC scores and *punishment rate* [95% C.I.: −0.72 to −0.08; *r* = −0.461, *p* = 0.02, Fisher's *Z*_r_ = −0.497 Figure 2A]. To further investigate whether empathic concern has an influence on decision speed in both help and punishment trials, we correlated IRI_EC and the difference in reaction times between help and punishment trials (i.e., RT_help-punish), finding a negative relationship [95% C.I.: −0.69 to −0.01; *r* = −0.406, *p* = 0.044, Fisher's *Z*_r_ = −0.431; Figure 2B].

A One-Way repeated measure ANOVA on the perceived unfairness rating of the offers showed a main effect of inequity level [95% C.I.: 5.09–5.51; *F*_(5, 120)_ = 225.967, *p* < 0.001, partial η^2^ = 0.904]. *Post-hoc* analyses revealed that ratings increased with the level of inequity of the offers (50/50: *M* = 1.48, SD = 1.12; 60/40: *M* = 3.52, SD = 1.30; 70/30: *M* = 5.24, SD = 0.93; 80/20: *M* = 6.24, SD = 0.93; 90/10: *M* = 7.32, SD = 0.48; 100/0: *M* = 8.00, SD = 0.00; *p* < 0.05, *Bonferroni* corrected).

### Imaging findings

#### Neural correlates of third-party help and punishment

**Figure 3:**
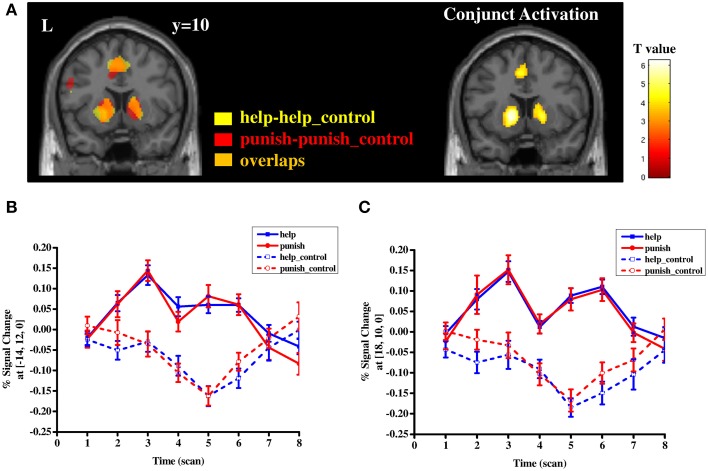
**Separate and conjunction mapping of regions involved in third-party help and punishment (A) and timecourse of percent signal change in the local peak voxel of left striatum (B) and right striatum (C) in four conditions (i.e., help, help_control, punish, punish_control)**. Error bars: SEM.

Both contrast *help* vs. *help_control* and *punish vs. punish_control* showed significant activation in several regions, including bilateral striatum, supplementary motor area/mid-cingulate cortex (BA 4/6), inferior/superior parietal lobule (BA 39/40) as well as visual areas (BA 17/18/19) (Table S1 and Figure 3). The conjunction analyses further confirmed that the bilateral striatum along with other areas mentioned above were activated by both contrasts, indicating that help- and punish-related cognitive processes shared some common neural bases (Table S1 and Figure 3). Activity in the bilateral striatum remained significant when controlling for motor responses due to button pressing (Table S2 and Figure S1). To test the differential neural correlates between these two processes, help and punishment decisions were directly contrasted, which yielded no significant difference in both directions. These results remained unchanged when controlling for fairness levels and transfer amounts (Tables S3, S4).

#### Relationship between empathic concern and brain activation during third-party decisions

**Figure 4:**
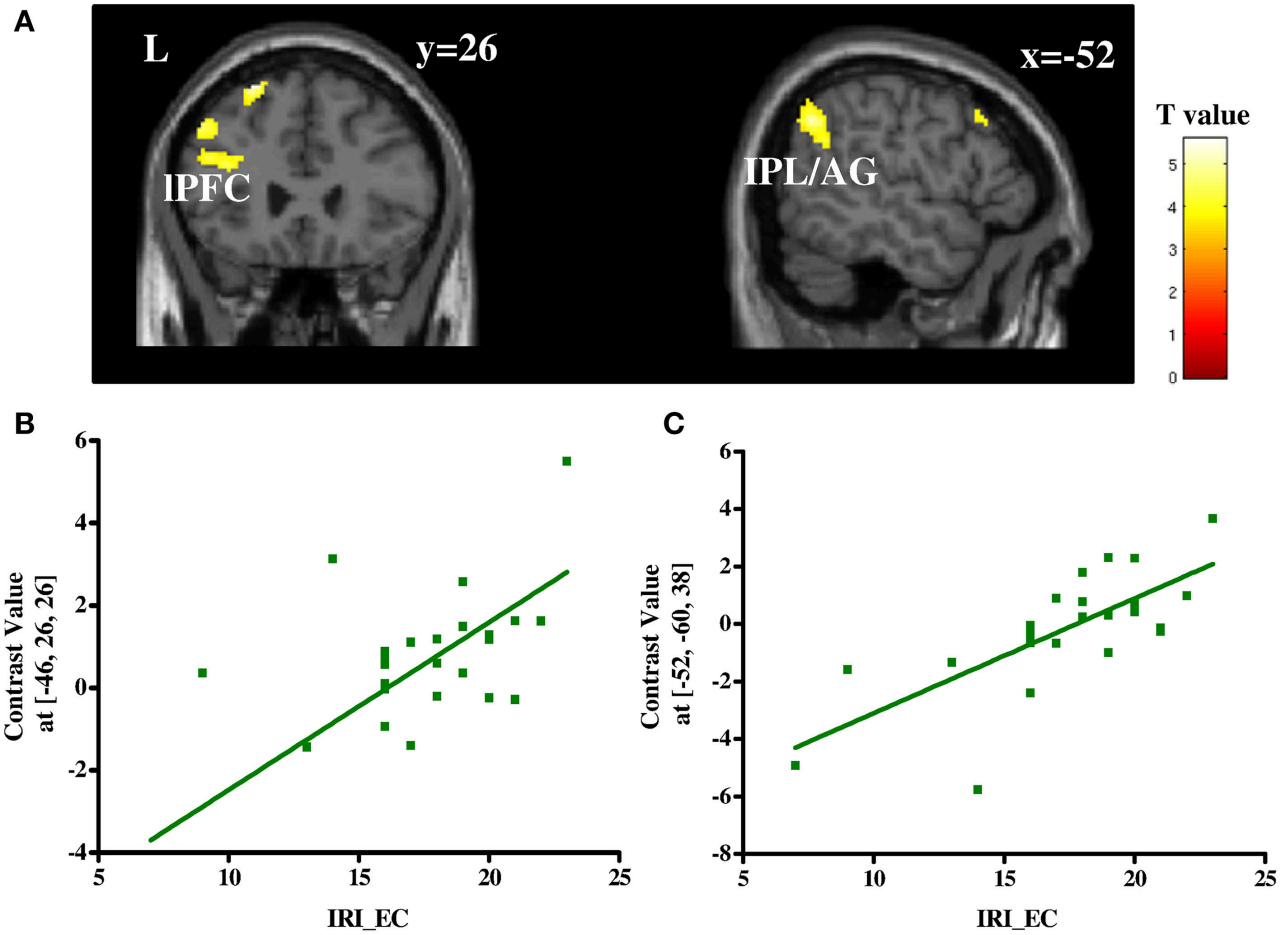
**Correlation between the contrast of help vs. punish and IRI_EC scores (A) and plots of the positive correlation between IRI_EC scores and contrast values in local peak voxel of left lPFC (B) and that of left IPL/AG (C)**. IRI_EC, empathic concern subscale of interpersonal reactivity index scale; lPFC, lateral prefrontal cortex; IPL, inferior parietal lobule; AG, angular gyrus.

To determine regions in which a change of the BOLD signal to third-party decisions varied with individual difference in trait empathy, a correlation analysis was performed between the contrast help vs. punishment and IRI_EC scores. Stronger positive correlations were detected in fronto-parietal regions including left lateral prefrontal cortex (lPFC, BA 9) and left angular gyrus/inferior parietal lobule (IPL/AG, BA 7/40; Table S5 and Figure 4). No negative correlations were found under the same threshold.

#### Functional connectivity pattern of third-party decisions

**Figure 5:**
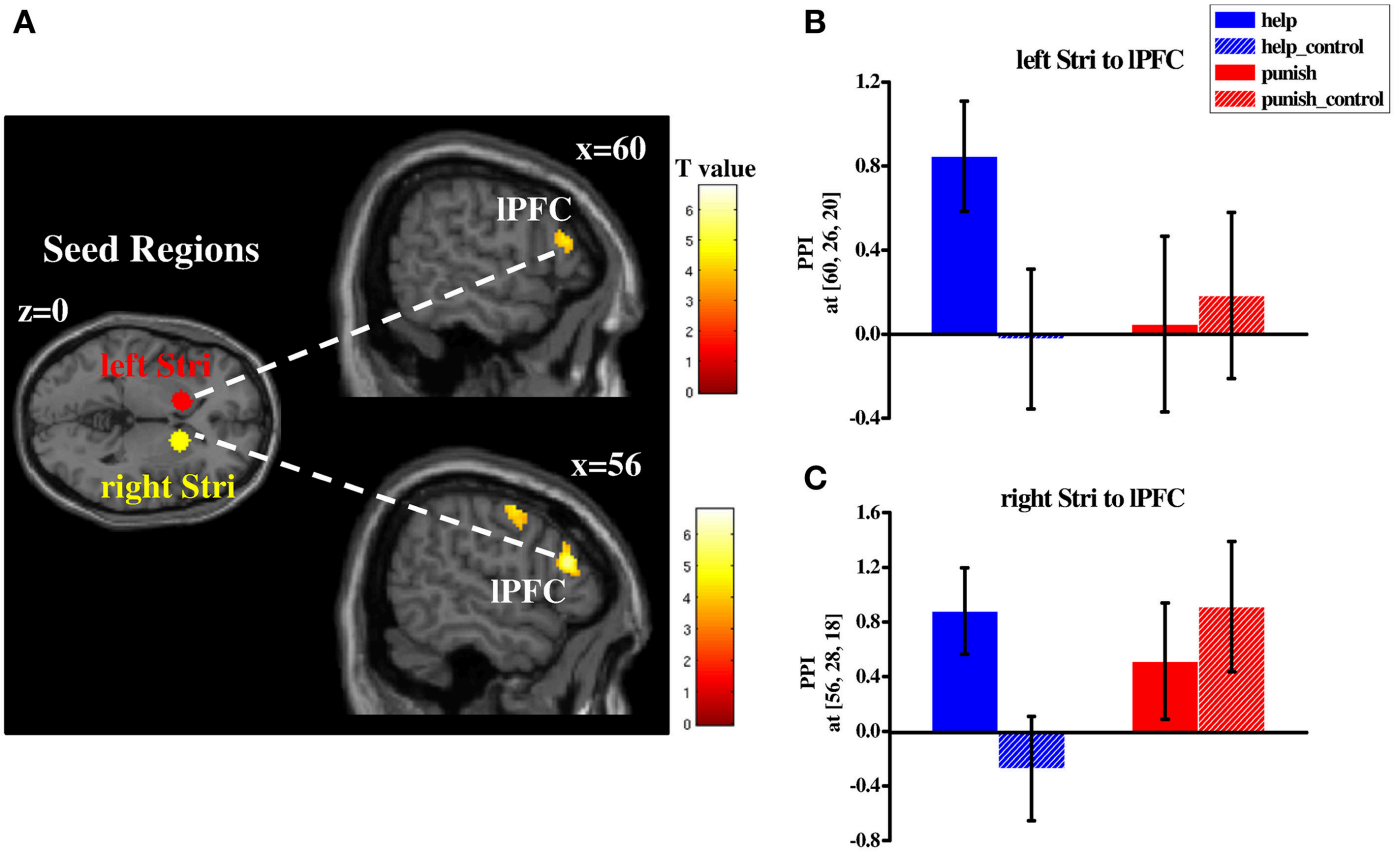
**Regions showing increased functional connectivity with bilateral striatum during third-party help decisions (compared with control conditions; (A) and plots of parameter estimates of PPI in the local peak voxel of right lPFC with left (B)/right (C) striatum in four conditions (i.e., help, help_control, punish, punish_control)**. Abbreviations: PPI, psycho-physiological interaction; lPFC, lateral prefrontal cortex; Stri, striatum; Error bars: SEM.

**Figure 6:**
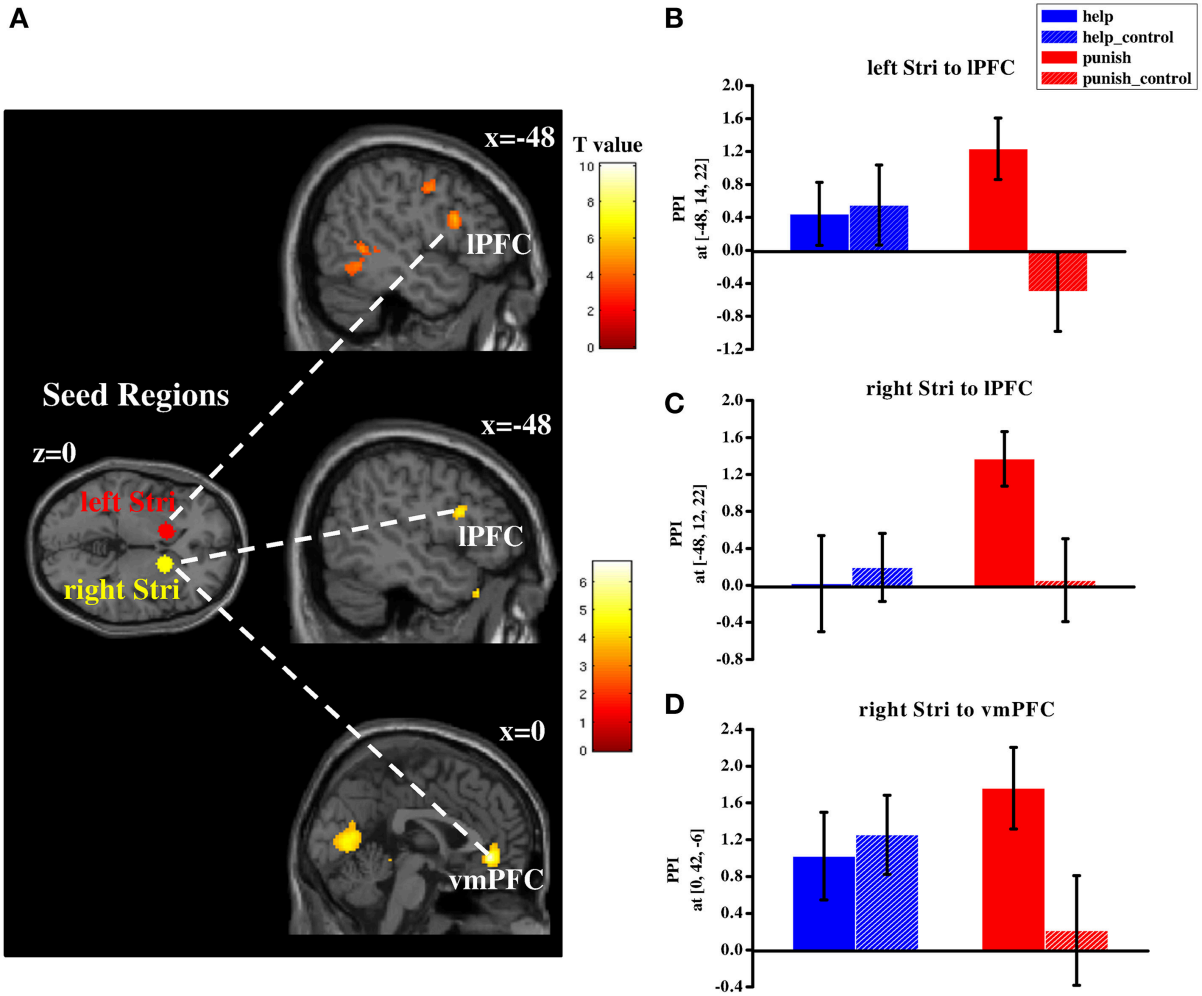
**Regions showing increased functional connectivity with bilateral striatum during third-party punishment decisions (compared with control conditions; (A) and plots of parameter estimates of PPI in local peak voxel of left lPFC with left (B)/right (C) striatum and that of vmPFC with right striatum (D) in four conditions (i.e., help, help_control, punish, punish_control)**. PPI, psycho-physiological interaction; lPFC, lateral prefrontal cortex; vmPFC, ventral medial prefrontal cortex; Stri, striatum; Error bars: SEM.

In order to investigate whether different networks are involved in third-party help and punishment a PPI analysis was conducted. Based on our hypotheses and the results of the conjunction analyses the striatum was used as the seed region (i.e., left and right striatum). PPI analyses were conducted during help and punishment decisions, respectively (both compared with their respective control conditions). Right lPFC (BA 45/46) showed increased functional connectivity with bilateral striatum during help decisions (Table S6 and Figure 5), whereas left lPFC (BA 44/45) showed enhanced functional connectivity with both seed regions during punishment decisions (Table S6 and Figure 6). Furthermore, ventral medial prefrontal cortex (vmPFC; BA 10/11/32) was observed to show increased connectivity only with right striatum when participants chose to punish (Table S6 and Figure 6). No significant difference in functional connectivity was found in a direct comparison of help and punishment decisions with either left or right striatum.

## Discussion

Our results reveal that both third-party help and third-party punishment share a common neuronal basis, but that specific networks are additionally involved in the two processes. The bilateral striatum was activated by both helping and punishing; functional connectivity between the bilateral striatum and the right lateral prefrontal cortex (lPFC) was increased during help and with left lPFC and ventromedial prefrontal cortex (vmPFC) during punishment. Individual differences in empathic concern correlated with people's preference to help or to punish. People with high empathic concern helped more frequently, were faster in their decision and showed higher activation in fronto-parietal regions during decisions to help.

The conjunction analysis indicated that third-party help and third-party punishment both share some common neural bases. In line with previous findings the striatum showed increased activation during altruistic help (Harbaugh et al., 2007; Hein et al., 2010; Genevsky et al., 2013) as well as during altruistic punishment (De Quervain et al., 2004). Helping friends or even strangers and punishing norm violators has been associated with activity in the striatum. However, so far striatal activation was only observed in third-party helping and second-party punishment paradigms, for example, while an investor chose to punish an untrustworthy trustee (De Quervain et al., 2004). This is to our knowledge the first study investigating third-party helping and punishing in the same study and showing that both are associated with striatal activation. The striatum is part of the human reward system, known to be activated by recognizing and evaluating rewards and learning from them (Bhanji and Delgado, 2014). Our results are in line with literature on charitable donation and second-party punishment suggesting that both helping an unknown person and punishing an offender is intrinsically rewarding (Fehr and Camerer, 2007; Harbaugh et al., 2007). Here we show that punishing an offender as a third person seems to be rewarding as well.

However, an alternative interpretation of this result cannot be ruled out completely. Participants were not required to response during the computer (control) trials in order to avoid additional cognitive (e.g., conflict) or affective (e.g., anger, frustration) processes. Unfortunately this paradigm thereby introduced a potential motor confound for the contrasts between help or punish decisions (button presses) and their corresponding control trials (no button presses). Besides its role in reward processing or representation of affective value, the striatum is also frequently associated with motor-related functions (Witt et al., 2008; Filevich et al., 2012; Guitart-Masip et al., 2012, 2014). In a recent study on the role of the striatum in decision making, Guitart-Masip and colleagues independently manipulated both action (i.e., “go” or “no go”) and valence (i.e., “to win” or “to avoid losing”) in an instrumental learning paradigm. They found that activity in the striatum reflected primarily the action requirements, independent of the valence of decisions (Guitart-Masip et al., 2012). This result suggests an involvement of the striatum in motivated action during decision making. In order to control for this, we performed an additional analysis in which the onset of the button presses were added in to the GLM as an independent regressor. This analysis showed that the bilateral striatum was still strongly activated during both third-party help and punishment even after controlling for the effect of button pressing (see Table S2 and Figure S1), indicating that activity in the striatum detected in the contrasts of third-party altruistic decisions and control trials is not likely driven by pure motor effects only. Rather it more likely reflects processes related to decision making, like rewarding processes as suggested by previous findings on altruistic decisions (e.g., charity donation, second-/third-party punishment De Quervain et al., 2004; Harbaugh et al., 2007; Hein et al., 2010; Strobel et al., 2011; Genevsky et al., 2013) and on reward processing (Haber and Knutson, 2010). However, since the onset of button pressing is not a random event as it is collinear to the onset of decision trials, the analysis unfortunately might not completely tease apart the effect of button pressing and that of decision processes. Since we cannot perfectly disentangle brain activity due to decision processes and due to motor processes (button press), the joint activation in striatum during third-party help and punishment decisions should be cautiously interpreted as reward-relevant processing.

Furthermore, our functional connectivity results suggest that besides the common neural basis, different networks are involved in third-party help and third-party punishment. Increased functional connectivity was found between the bilateral striatum and right lPFC during help decisions whereas left lPFC and the bilateral striatum showed increased functional connectivity during punishment decisions. Furthermore, vmPFC showed increased connectivity with right striatum when participants chose to punish. Generally, our PPI findings are consistent with the anatomical connectivity of the striatum, which was found to be connected with both lateral and ventral/medial parts of the prefrontal cortex (Haber and Knutson, 2010). Specifically, lPFC is known to be engaged in cognitive/executive control and goal-directed decisions (Miller and Cohen, 2001; Tanji and Hoshi, 2008). In the social-economic domain, especially the right lPFC was shown to be involved in the control of selfish impulses (Knoch et al., 2006; Ruff et al., 2013; Strang et al., 2014). For example, disrupting the right lPFC via low-frequency repetitive TMS caused people to make riskier decisions (Knoch and Fehr, 2007) and to exhibit more norm violating behaviors (Strang et al., 2014). A recent TMS study found that people show more impulsive behavior in an inter-temporal choice task while the left lPFC was inhibited (Figner et al., 2010). Intriguingly, left lPFC showed stronger activity when participants chose to costly punish proposers as a third-party compared to as a second-party, indicating that cognitive-control processes, instead of revenge-driven motives, are involved in third-party punishment (Strobel et al., 2011). Consistently, a behavioral study showed that punishment of free-riders by cooperators is linked to self-control abilities (Espín et al., 2012). Moreover, some studies showed an increased functional connectivity between lPFC and striatum while people controlled reward-related responses to food cues (Hare et al., 2011) or monetary reward (Delgado et al., 2008). Since help and punishment are costly in our paradigm, both require control of selfish impulses in order to engage in one of the two behaviors. Hence, it is possible that in our paradigm increased connectivity between lPFC and bilateral striatum during help and punishment decisions is due to these control processes. Another region found to have increased connectivity with the striatum is the vmPFC, which has been shown to be involved in a variety of cognitive and affective processes including integrating emotional information (Naqvi et al., 2006) and subjective valuation during decision making (Ruff and Fehr, 2014). Interestingly, increased activity in the vmPFC was also found when choosing to costly punish an untrustworthy trustee (De Quervain et al., 2004). Our findings seem to support the view of a potentially stronger involvement of the vmPFC in third-party punishment rather than help.

Nevertheless, two issues limit our interpretation on the PPI results. Firstly, it is important to mention that there was no significant difference between the functional connectivity during help and punishment decisions when directly contrasting the connectivity results in both decisions, which weakens our inference about differential neural networks involved in each decision. This might be due to insufficient sample size or inadequate numbers of trials in each condition. Secondly, as both PPI analyses are based on the corresponding control trials (help vs. help_control or punish vs. punish_control), the results are also influenced by the motor confound mentioned above. Thus, the connectivity pattern might also reflect a motor effect during both decisions compared with the pure observation in the control trials. Since the PPI analyses are rather explorative, further research is needed to shed more light on the network involved in third-party help and punishment.

Moreover, our results demonstrate that individual differences in empathic concern influence our decision to help or to punish on a behavioral as well as on a neural level. People with high levels of empathic concern chose to help more frequently, were faster in their decision to help and showed higher activation in frontoparietal regions (i.e., left lPFC and left IPL/AG) during this decision. The behavioral findings are in line with previous research (Leliveld et al., 2012), in which the authors also reported that people with high empathic concern prefer to help instead of punishing, whereas people with low empathic concern prefer to punish instead of helping. In addition our results demonstrate that high empathic people are also faster in deciding to help compared to deciding to punish, whereas people with low empathic concern show the reversed pattern; they are faster in deciding to punish instead of helping. Faster reaction times are often interpreted as a sign of less conflict between the options someone has to choose from and less cognitive processing (Rand et al., 2012). According to this literature the results suggest that for high empathic people deciding to help needs less cognitive processing. For them the decision to either help or to punish does not involve a conflict, help is the default option for them. Low empathic people also do not encounter a conflict when deciding between help and punishment, their default option is to punish. Whether someone helps a victim or punishes the offender hence depends on how much empathic concern someone has. Both regions correlating with empathic concern, lPFC and IPL/AG, are considered as the core components of the frontoparietal network (FPN), which play an important role in top-down cognitive control and attention (Corbetta and Shulman, 2002; Dosenbach et al., 2008). Gromet and Darley (2009) argue that punishment might be the default choice after observing injustice until people are asked to focus on the victim. Without explicit requirements to focus on the victim, such an attention shift might be influenced by individual's personality trait, in this case empathic concern. Our results hint towards such an empathy-based attention shift. However, this interpretation is inconsistent with the reaction time findings, which suggests that help is the default for people with high empathic concern. Thus, the role of FPN in mediating the relationship between empathic concerns and the two altruistic decisions still needs further investigation. Future studies might shed more light on this question by adopting other techniques (such as eye-tracking) to investigate the difference in fine-grained information search patterns between high and low empathic people during deciding to either help or punish.

There are several limitations of this study. One constraint is the difference in motor demands between the decision and control trials as mentioned above. Future studies should try to find a clearer way to disentangle activity due to the decision process and motor responses. Another limitation is the high number of excluded participants. We were only able to use data from 25 out of 36 participants, because ten participants did not show enough variability in their behavior to define all necessary regressors. Since trials were sorted into different conditions according to participant's behavior in the corresponding trial, sufficient numbers of trials (>25) for one condition in order to calculate a contrast cannot be guaranteed. Although 25 participants is still a widely accepted sample size in the field of cognitive neuroimaging, statistical power might explain the non-significant difference especially for the PPI results. Since people who exhibit either very high or very low empathic concerns have a preference for either helping or punishing, respectively, they show less variability in their decisions on the individual level. One possibility to minimize dropout rates is to increase the variability in decision behavior by only inviting participants with empathic concern score in the medium range and thereby increasing statistical power. Additionally a pre-screening could be used to exclude participants who are very selfish and are not willing to help or punish at all.

Taken together, by using a modified third-party decision paradigm with fMRI, our study provides first evidence for the neural basis of third-party help and punishment decisions. Both altruistic decisions activated bilateral striatum, indicating that intrinsic reward processes are involved in both third-party help and punishment decisions. Differential functional connectivity networks during third-party help and punishment suggest different cognitive processes underlying both decisions. Moreover, the present study replicated previous behavioral findings on the role of empathic concern in mediating people's decisions to either help or punish. Further its underlying neural correlates in frontoparietal regions were detected. Despite some limitations, these findings may provide insights for better understanding the mechanism underlying altruism and social norm enforcement.

### Conflict of interest statement

The authors declare that the research was conducted in the absence of any commercial or financial relationships that could be construed as a potential conflict of interest.
