# Supplementary material for: Helping or punishing strangers: neural correlates of altruistic decisions as third-party and of its relation to empathic concern
Source: Front Behav Neurosci. 2015 Feb 18;9:24. doi: 10.3389/fnbeh.2015.00024 (PMC4332347; doi:10.3389/fnbeh.2015.00024)
Supplement: Supplementary file 1 [file Table1.DOCX]

***Supplementary Material***

**Helping or punishing strangers: neural correlates of altruistic decisions as third-party and of its relation to empathic concern**

**Yang Hu^1*†^, Sabrina Strang^1,2 †^, Bernd Weber^1,3^**

^1^Center for Economics and Neuroscience, University of Bonn, Bonn, Germany

^2^Department of Psychology, University of Lübeck, Germany

^3^Department of Epileptology, University Hospital Bonn, Bonn, Germany

*** Correspondence:** Yang Hu, Center for Economics and Neuroscience, University of Bonn, Nachtigallenweg 86, Bonn, 53127, Germany.

[huyang@uni-bonn.de](mailto:huyang@uni-bonn.de)

^†^These authors are co-first authors.

1. **Supplementary Figures and Tables**

## Suplementary Tables

**Supplementary Table 1. Brain activation of third-party help and punishment decisions.** Note: threshold is set to p < 0.001, k=50, uncorrected; * refers to clusters survived at p < 0.05, FWE corrected; L=left, R=right, B=bilateral; brain regions are labeled according to the automated anatomic labeling toolbox for SPM8.

| Brain Region | Hemisphere | Cluster Size | MNI Coordinates | | | BA | T-value |
| --- | --- | --- | --- | --- | --- | --- | --- |
|  |  |  | x | y | z |  |  |
| HELP > HELP_CONTROL | |  |  |  |  |  |  |
| Middle Frontal Gyrus | L | 93 | -46 | 36 | 22 | 46 | 4.16 |
| Middle Frontal Gyrus | R | 147 | 40 | 48 | 8 | 46 | 5.20 |
| Anterior Cingulate Gyrus/ Supplementary Motor Area | B | 937 | -4 | 12 | 42 | 6/24/32 | 6.92* |
| Insula/Superior Temporal Gyrus | R | 254 | 46 | -18 | 10 | 13/22 | 6.90* |
| Precentral Gyrus/ Postcentral Gyrus/Inferior Parietal Lobule/Superior Parietal Gyrus | L | 2877 | -46 | -2 | 58 | 1/2/3/6/  7/39/40 | 8.93* |
| Precental Gyrus/ Postcentral Gyrus/Inferior Parietal Lobule/Superior Parietal Gyrus | R | 2301 | 26 | -64 | 58 | 1/2/3/4/  7/39/40 | 7.31* |
| Inferior/Middle Occipital Gyrus | L | 1438 | -38 | -78 | 0 | 17/18/19 | 7.56* |
| Inferior/Middle Occipital Gyrus | R | 1760 | 34 | -84 | 2 | 17/18/19 | 8.85* |
| Caudate/Putamen | L | 574 | -14 | 14 | 4 |  | 8.35* |
| Caudate/Putamen | R | 264 | 16 | 14 | -2 |  | 7.71* |
|  |  |  |  |  |  |  |  |
| PUNISH > PUNISH_CONTROL | |  |  |  |  |  |  |
| Supplementary Motor Area/Midcingulate Gyrus/ Anterior Cingulate Gyrus | B | 1167 | 10 | -8 | 50 | 6/24/31/32 | 6.71* |
| Precentral Gyrus/ Postcental Gyrus/Inferior Parietal Lobule/Superior Parietal Lobule | L | 1870 | -40 | -38 | 44 | 2/3/4/  7/39/40 | 7.22* |
| Precentral Gyrus/ Postcental Gyrus | R | 1047 | 48 | -18 | 50 | 2/3/4 | 6.74* |
| Superior Temporal Gyrus/ Insula | L | 206 | -50 | -34 | 8 | 13/41 | 4.68* |
| Inferior Occipital Gyrus/ Middle Occipital Gyrus/ Middle Temporal Gyrus | L | 573 | -44 | -72 | 6 | 17/18/  19/37 | 5.55* |
| Inferior Occipital Gyrus/ Middle Occipital Gyrus/ Middle Temporal Gyrus | R | 629 | 46 | -66 | 2 | 17/18/  19/37 | 7.08* |
| Caudate/Putamen | L | 599 | -16 | 10 | -2 |  | 7.48* |
| Caudate/Putamen | R | 255 | 24 | -12 | 2 |  | 7.26* |
|  |  |  |  |  |  |  |  |
| CONJUNCTION | |  |  |  |  |  |  |
| Caudate/Putamen | L | 382 | -16 | 12 | 0 |  | 6.26* |
| Caudate/Putamen | R | 250 | 16 | -20 | 6 |  | 6.08* |
| Precentral Gyrus/ Postcentral Gyrus/Inferior Parietal Lobule/Superior Parietal Lobule | L | 1493 | -38 | -38 | 38 | 2/3/4/  6/40 | 5.13* |
| Precentral Gyrus/ Postcentral Gyrus | R | 922 | 40 | -12 | 58 | 2/3/4/6 | 5.69* |
| Middle Temporal Gyrus/ Middle Occipital Gyrus | L | 125 | -44 | -70 | 6 | 19/37 | 5.05 |
| InferiorTemporal Gyrus/ Middle Temporal Gyrus | R | 236 | 46 | -66 | 4 | 19/37 | 5.57* |
| Supplementary Motor Area/Midcingulate Gyrus/Anterior Cingulate Gyrus | M | 583 | -4 | 14 | 46 | 6/9/24/32 | 4.97* |
| Superior Temporal Gyrus/ Insula/Postcentral Gyrus | L | 324 | -38 | -34 | 16 | 13/41/42 | 4.16* |
| Superior Temporal Gyrus/ Insula | R | 232 | 50 | -14 | 10 | 13/22/41 | 4.56* |
| Inferior Occipital Gyrus/ Middle Occipital Gyrus | L | 96 | -26 | -92 | -4 | 18 | 4.10 |
| Inferior Occipital Gyrus/ Middle Occipital Gyrus | R | 130 | 30 | -86 | -2 | 18/19 | 4.28 |
