# Supplementary material for: Helping or punishing strangers: neural correlates of altruistic decisions as third-party and of its relation to empathic concern
Source: Front Behav Neurosci. 2015 Feb 18;9:24. doi: 10.3389/fnbeh.2015.00024 (PMC4332347; doi:10.3389/fnbeh.2015.00024)
Supplement: Supplementary file 2 [file Table2.DOCX]

***Supplementary Material***

**Helping or punishing strangers: neural correlates of altruistic decisions as third-party and of its relation to empathic concern**

**Yang Hu^1*†^, Sabrina Strang^1,2 †^, Bernd Weber^1,3^**

^1^Center for Economics and Neuroscience, University of Bonn, Bonn, Germany

^2^Department of Psychology, University of Lübeck, Germany

^3^Department of Epileptology, University Hospital Bonn, Bonn, Germany

*** Correspondence:** Yang Hu, Center for Economics and Neuroscience, University of Bonn, Nachtigallenweg 86, Bonn, 53127, Germany.

[huyang@uni-bonn.de](mailto:huyang@uni-bonn.de)

^†^These authors are co-first authors.

1. **Supplementary Figures and Tables**

## Supplementary Tables

**Supplementary Table 2. Brain activation of third-party help and punishment decisions after controlling the effect of button pressing during decision.** Note: In this GLM, we added the onset of button presses to control for motor related activity, with the other regressors being the same as the GLM reported in the main manuscript; one-sample T-test was used for the group analysis; threshold is set to p < 0.001, k=50, uncorrected; * refers to clusters survived at p < 0.05, FWE corrected; L=left, R=right, B=bilateral; brain regions are labeled according to the automated anatomic labeling toolbox for SPM8.

| Brain Region | Hemisphere | Cluster Size | MNI Coordinates | | | BA | T-value |
| --- | --- | --- | --- | --- | --- | --- | --- |
|  |  |  | x | y | z |  |  |
| HELP>HELP_CONTROL |  |  |  |  |  |  |  |
| Postcentral Gyrus/Precentral Gyrus | L | 355 | -46 | -28 | 64 | 1/3/4/6 | 6.03* |
| Inferior Parietal Gyrus/Postcentral Gyrus/Precentral Gyrus | R | 602 | 58 | -30 | 54 | 1/2/3/4/  6/40 | 5.56* |
| Caudate/Putamen/Pallidum | L | 274 | -12 | 14 | 2 |  | 5.87* |
| Caudate/Putamen/Pallidum | R | 225 | 16 | 12 | 0 |  | 6.98* |
| Thalumus | L | 158 | 16 | -16 | 4 |  | 5.15 |
| Thalumus | R | 162 | -18 | -12 | 6 |  | 4.73 |
|  |  |  |  |  |  |  |  |
| PUNISH>PUNISH_CONTROL |  |  |  |  |  |  |  |
| Supplemental Motor Area | R | 90 | 16 | -8 | 52 | 24 | 5.17 |
| Postcentral Gyrus/Precentral Gyrus | L | 240 | -36 | -22 | 48 | 3/4 | 4.79* |
| Precentral Gyrus/Postcentral Gyrus | R | 353 | 38 | -12 | 54 | 3/4/6 | 5.83* |
| Caudate/Putamen/Pallidum/  Thalamus/Brainstem | B | 1766 | 6 | -28 | -10 |  | 7.37* |
|  |  |  |  |  |  |  |  |
| CONJUNCTION |  |  |  |  |  |  |  |
| Putamen/Pallidum/Caudate | L | 205 | -14 | 10 | 0 |  | 4.81 |
| Pallidum/Putamen/Caudate | R | 126 | 18 | 6 | 0 |  | 4.42 |
| Postcentral Gyrus/Precentral Gyrus | L | 195 | -50 | -20 | 54 | 3/4 | 3.99 |
| Precentral Gyrus/Postcentral Gyrus | R | 196 | 38 | -14 | 60 | 3/4/6 | 4.50 |
| Thalamus/Brainstem | B | 657 | -2 | -24 | -12 |  | 5.27* |
|  |  |  |  |  |  |  |  |
| BUTTON PRESS |  |  |  |  |  |  |  |
| Superior/Middle Frontal Gyrus | R | 185 | 32 | -4 | 66 | 6 | 5.14 |
| Precentral Gyrus | R | 72 | 36 | -10 | 42 | 6 | 4.25 |
| Inferior/Middle/Superior Occipital Gyrus/  Fusiform Gyrus/  Precuneus/Cuneus/Precentral Gyrus/Postcentral Gyrus/  Inferior/Superior Parietal Gyrus/  Inferior/Superior Temporal Gyrus/  Supplementary Motor Area/  Anterior/Posterior Cingulate Gyrus/  Middle Frontal Gyrus/ Insula/  Parahippocampa Gyrus/  Putamen/Caudate/Cerebellum | B | 66552 | 22 | -78 | -14 | 3/4/5/6/  7/8/9/10/  13/18/19/  20/22/23/  24/30/32/  37/38/39/  40/42/43/  44/45/  46/47 | 15.64* |
