# Supplementary material for: Helping or punishing strangers: neural correlates of altruistic decisions as third-party and of its relation to empathic concern
Source: Front Behav Neurosci. 2015 Feb 18;9:24. doi: 10.3389/fnbeh.2015.00024 (PMC4332347; doi:10.3389/fnbeh.2015.00024)
Supplement: Supplementary file 3 [file Table3.DOCX]

***Supplementary Material***

**Helping or punishing strangers: neural correlates of altruistic decisions as third-party and of its relation to empathic concern**

**Yang Hu^1*†^, Sabrina Strang^1,2 †^, Bernd Weber^1,3^**

^1^Center for Economics and Neuroscience, University of Bonn, Bonn, Germany

^2^Department of Psychology, University of Lübeck, Germany

^3^Department of Epileptology, University Hospital Bonn, Bonn, Germany

*** Correspondence:** Yang Hu, Center for Economics and Neuroscience, University of Bonn, Nachtigallenweg 86, Bonn, 53127, Germany.

[huyang@uni-bonn.de](mailto:huyang@uni-bonn.de)

^†^These authors are co-first authors.

1. **Supplementary Figures and Tables**

## Suplementary Tables

**Supplementary Table 3. Brain activation of third-party help and punishment decisions after controlling the effect of unfairness.** Note: In this GLM, we added the unfairness level (i.e. 60/40, 70/30, 80/20, 90/10, 100/0; they have been transformed to 1, 2, 3, 4, 5) as parametric modulators for the following regressors: “help” (with “help_para”), “punish” (with “punish_ para”), “help_control” (with “help_control_ para”), “punish_control” (with “punish_control_ para”), with the other regressors being the same as the GLM reported in the manuscript; one-sample T-test was used for the group analysis; threshold is set to p < 0.001, k=50, uncorrected; * refers to clusters survived at p < 0.05, FWE corrected; L=left, R=right, B=bilateral; brain regions are labeled according to the automated anatomic labeling toolbox for SPM8.

| Brain Region | Hemisphere | Cluster Size | MNI Coordinates | | | BA | T-value |
| --- | --- | --- | --- | --- | --- | --- | --- |
|  |  |  | x | y | z |  |  |
| HELP > HELP_CONTROL |  |  |  |  |  |  |  |
| Middle Frontal Gyrus | L | 92 | -46 | 36 | 22 | 46 | 4.18 |
| Middle Frontal Gyrus | R | 141 | 40 | 48 | 8 | 10 | 5.23 |
| Supplementary Motor Area/  Middle Cingulate Cortex | B | 933 | -4 | 12 | 42 | 6/24/32 | 6.97* |
| Posterior Gyrus/  Paracentral Lobule | L | 125 | -16 | -30 | 76 | 2/3/4 | 6.09 |
| Posterior Insula/  Superior Temporal Gyrus | R | 255 | 46 | -18 | 10 | 13/22/41 | 6.91* |
| Precentral Gyrus/Postcentral Gyrus/Inferior Parietal Lobule/Superior Parietal Gyrus | B | 5908 | -34 | -46 | 44 | 1/2/3/4/6/  7/39/40 | 8.67* |
| Inferior/Midddle Occipital Gyrus | B | 3204 | 34 | -84 | 2 | 17/18/19 | 8.87* |
| Caudate/Putamen | L | 445 | -12 | 12 | 4 |  | 8.28* |
| Caudate/Putamen | R | 451 | 16 | 12 | -2 |  | 7.64* |
|  |  |  |  |  |  |  |  |
| PUNISH > PUNISH_CONTROL | |  |  |  |  |  |  |
| Anterior Cingulate Cortex | R | 58 | 12 | 24 | 28 | 32 | 4.05 |
| Precentral Gyrus/  Inferior Frontal Gyrus | L | 167 | -52 | 4 | 24 | 6/9 | 4.74 |
| Middle/Superior Temporal Gyrus/Posterior Insula | L | 320 | -50 | -34 | 8 | 13/41 | 4.79* |
| Precentral Gyrus/Postcentral Gyrus/Inferior Parietal Lobule/Superior Parietal Gyrus/Supplementary Motor Area/Middle Cingulate Gyrus | B | 5205 | -40 | -38 | 44 | 1/2/3/4/6/7/  24/32/39/40 | 7.45* |
| Inferior/Middle Occipital Gyrus/Middle Temporal Gyrus | L | 653 | -44 | -72 | 6 | 18/19/37 | 5.62* |
| Inferior/Middle Occipital Gyrus/Middle Temporal Gyrus | R | 946 | 46 | -66 | 2 | 18/19/37 | 7.16* |
| Caudate/Putamen | L | 584 | -16 | 8 | -2 |  | 7.45* |
| Caudate/Putamen | R | 230 | 18 | -6 | -2 |  | 7.30* |
|  |  |  |  |  |  |  |  |
| CONJUNCTION |  |  |  |  |  |  |  |
| Caudate/Putamen | L | 376 | -16 | 12 | 0 |  | 6.21* |
| Caudate/Putamen | R | 253 | 16 | 14 | 2 |  | 4.891* |
| Supplementary Motor Area/  Mid-cingulate Gyrus | B | 655 | -4 | 14 | 46 | 6/24/32 | 4.99* |
| Precentral Gyrus | L | 78 | -52 | 4 | 34 | 60/90 | 3.86 |
| Precentral/Postcentral Gyrus | R | 995 | 40 | -12 | 58 | 2/3/4/6 | 5.73* |
| Superior Temporal Gyrus/Insula | L | 367 | -36 | -32 | 16 | 40/41 | 4.18* |
| Superior Temporal Gyrus/Insula | R | 234 | 48 | -16 | 10 | 13/42 | 4.50* |
| Precentral/Postcentral Gyrus/  Inferior Parietal Lobule | L | 1704 | -38 | -38 | 38 | 2/3/4/40 | 5.26* |
| Middle Occipital Gyrus | L | 138 | -44 | -70 | 6 | 19/37 | 5.09 |
| Middle Temporal Gyrus/  Middle Occipital Gyrus | R | 305 | 46 | -66 | 4 | 37 | 5.64* |
| Inferior/Middle Occipital Gyrus | L | 111 | -26 | -92 | -4 | 18 | 4.2 |
| Inferior/Middle Occipital Gyrus | R | 184 | 30 | -86 | -2 | 18 | 4.36 |
|  |  |  |  |  |  |  |  |
| HELP_PARA: POSITIVE |  |  |  |  |  |  |  |
| No significant region |  |  |  |  |  |  |  |
|  |  |  |  |  |  |  |  |
| HELP_ PARA: NEGATIVE |  |  |  |  |  |  |  |
| No significant region |  |  |  |  |  |  |  |
|  |  |  |  |  |  |  |  |
| PUNISH_ PARA: POSITIVE |  |  |  |  |  |  |  |
| No significant region |  |  |  |  |  |  |  |
|  |  |  |  |  |  |  |  |
| PUNISH_ PARA: NEGATIVE |  |  |  |  |  |  |  |
| Ligual Gyrus | R | 307 | 10 | -78 | -2 | 17/18 | 4.98 |
