# Supplementary material for: Helping or punishing strangers: neural correlates of altruistic decisions as third-party and of its relation to empathic concern
Source: Front Behav Neurosci. 2015 Feb 18;9:24. doi: 10.3389/fnbeh.2015.00024 (PMC4332347; doi:10.3389/fnbeh.2015.00024)
Supplement: Supplementary file 4 [file Table4.DOCX]

***Supplementary Material***

**Helping or punishing strangers: neural correlates of altruistic decisions as third-party and of its relation to empathic concern**

**Yang Hu^1*†^, Sabrina Strang^1,2 †^, Bernd Weber^1,3^**

^1^Center for Economics and Neuroscience, University of Bonn, Bonn, Germany

^2^Department of Psychology, University of Lübeck, Germany

^3^Department of Epileptology, University Hospital Bonn, Bonn, Germany

*** Correspondence:** Yang Hu, Center for Economics and Neuroscience, University of Bonn, Nachtigallenweg 86, Bonn, 53127, Germany.

[huyang@uni-bonn.de](mailto:huyang@uni-bonn.de)

^†^These authors are co-first authors.

1. **Supplementary Figures and Tables**

## Suplementary Tables

**Supplementary Table 4. Brain activation of third-party help and punishment decisions after controlling the effect of transfer amount (rate).** Note: In this GLM, we added the transfer amount as parametric modulators for the following regressors: “help” (with “help_para”), “punish” (with “punish_ para”), “help_control” (with “help_control_ para”), “punish_control” (with “punish_control_ para”), with the other regressors being the same as the GLM reported in the manuscript; one-sample T-test was used for the group analysis; threshold is set to p < 0.001, k=50, uncorrected; * refers to clusters survived at p < 0.05, FWE corrected; L=left, R=right, B=bilateral; brain regions are labeled according to the automated anatomic labeling toolbox for SPM8.

| Brain Region | Hemisphere | Cluster Size | MNI Coordinates | | | BA | T-value |
| --- | --- | --- | --- | --- | --- | --- | --- |
| HELP > HELP_CONTROL |  |  |  |  |  |  |  |
| Inferior/Middle Frontal Gyrus | L | 95 | -46 | 36 | 22 | 46 | 4.14 |
| Middle Frontal Gyrus | R | 139 | 40 | 48 | 8 | BA 10/46 | 5.16 |
| Supplementary Motor Area/  Middle Cingulate Cotex | B | 917 | -4 | 14 | 42 | 6/24/32 | 6.95* |
| Postcentral Fyrus/Paracentral Lobule | L | 129 | -16 | -30 | 76 |  | 7 |
| Insula/Superior Temporal Gyrus | R | 254 | 46 | -18 | 10 |  | 6.83 |
| Inferior/Superior Parietal Lobule/  Precentral Gyrus/Postcentral Gyrus | B | 5785 | -46 | -2 | 58 | 1/2/3/4/6/  7/31/40 | 9.09* |
| Inferior/Midddle Occipital Gyrus | B | 3184 | 34 | -84 | 2 | 17/18/19/37 | 8.89* |
| Caudate/Putamen | L | 452 | -12 | 12 | 4 |  | 8.34* |
| Caudate/Putamen | R | 457 | 16 | 12 | -2 |  | 7.60* |
|  |  |  |  |  |  |  |  |
| PUNISH > PUNISH_CONTROL |  |  |  |  |  |  |  |
| Anterior Cingulate Cortex | R | 55 | 12 | 24 | 28 | 32 | 4.14 |
| Precentral Gyrus/  Inferior Frontal Gyrus | L | 154 | -52 | 4 | 24 | 6/9 | 4.67 |
| Middle/Superior Temporal Gyrus/ Insula/  Postcentral Gyrus | L | 293 | -50 | -34 | 8 | 13/41 | 4,64* |
| Inferior/Superior Parietal Lobule/  Precentral Gyrus/Postcentral Gyrus/  Suppplementary Motor Area | B | 3755 | -40 | -38 | 44 | 1/2/3/4/6/  24/32/40 | 7,14* |
| Fusiform Gyrus | L | 104 | -38 | -44 | -30 | 37 | 4.65 |
| Fusiform Gyrus | R | 193 | 40 | -48 | -22 | 37 | 4.50 |
| Inferior/Middle Occipital Gyrus/  Middle Temporal Gyrus | L | 586 | -44 | -72 | 6 | 18/19/37 | 5,56* |
| Inferior/Middle Occipital Gyrus/  Middle Temporal Gyrus | R | 850 | 46 | -66 | 2 | 18/19/37 | 7,12* |
| Caudate/Putamen | L | 496 | -16 | 10 | -2 |  | 7,43* |
| Caudate/Putamen | R | 191 | 14 | 14 | 10 |  | 5.66 |
|  |  |  |  |  |  |  |  |
| CONJUNCTION |  |  |  |  |  |  |  |
| Caudate/Putamen | L | 384 | -16 | 12 | 0 |  | 6,26* |
| Caudate/Putamen | R | 247 | 16 | 14 | 2 |  | 4,86* |
| Supplementary Motor Area/  Middle Cingulate Gyrus | L | 646 | -4 | 14 | 46 | 24/32 | 4,99* |
| Precentral Gyrus/  Inferior Frontal Gyrus | L | 66 | -52 | 4 | 34 | 9 | 3.84 |
| Postcentral/Precentral Gyrus | R | 961 | 40 | -12 | 58 | BA 3/4 | 5,70* |
| Insula/Superior Temporal Gyrus/  Postcentral Gyrus | L | 345 | -38 | -34 | 16 | 13/22/41 | 4,16* |
| Insula/Superior Temporal Gyrus | R | 231 | 50 | -14 | 10 | 13/22 | 4,52* |
| Inferior/Superior Parietal Lobule/Postcentral Gyrus | L | 1660 | -38 | -38 | 38 | BA 3/40 | 5,14* |
| Middle Occipital Gyrus | L | 126 | -44 | -70 | 6 | 37 | 5.04 |
| Middle/Inferior Temporal Gyrus | R | 302 | 46 | -66 | 4 | 19/37 | 5,49* |
| Inferior Occipital Gyrus | L | 98 | -26 | -92 | -4 | 18 | 4.13 |
| Inferior Occipital Gyrus | R | 168 | 30 | -86 | -2 | 18 | 4.32 |
|  |  |  |  |  |  |  |  |
| HELP_PARA: POSITIVE |  |  |  |  |  |  |  |
| No significant region |  |  |  |  |  |  |  |
|  |  |  |  |  |  |  |  |
| HELP_ PARA: NEGATIVE |  |  |  |  |  |  |  |
| No significant region |  |  |  |  |  |  |  |
|  |  |  |  |  |  |  |  |
| PUNISH_ PARA: POSITIVE |  |  |  |  |  |  |  |
| No significant region |  |  |  |  |  |  |  |
|  |  |  |  |  |  |  |  |
| PUNISH_ PARA: NEGATIVE |  |  |  |  |  |  |  |
| Precentral Gyrus | L | 64 | -34 | -18 | 46 | 4 | 4.53 |
