# Supplementary material for: Helping or punishing strangers: neural correlates of altruistic decisions as third-party and of its relation to empathic concern
Source: Front Behav Neurosci. 2015 Feb 18;9:24. doi: 10.3389/fnbeh.2015.00024 (PMC4332347; doi:10.3389/fnbeh.2015.00024)
Supplement: Supplementary file 5 [file Table5.DOCX]

***Supplementary Material***

**Helping or punishing strangers: neural correlates of altruistic decisions as third-party and of its relation to empathic concern**

**Yang Hu^1*†^, Sabrina Strang^1,2 †^, Bernd Weber^1,3^**

^1^Center for Economics and Neuroscience, University of Bonn, Bonn, Germany

^2^Department of Psychology, University of Lübeck, Germany

^3^Department of Epileptology, University Hospital Bonn, Bonn, Germany

*** Correspondence:** Yang Hu, Center for Economics and Neuroscience, University of Bonn, Nachtigallenweg 86, Bonn, 53127, Germany.

[huyang@uni-bonn.de](mailto:huyang@uni-bonn.de)

^†^These authors are co-first authors.

1. **Supplementary Figures and Tables**

## Suplementary Tables

**Supplementary Table 5. Correlation between brain activation of the contrast of help vs. punishment and IRI_EC scores.** Note: threshold is set to p < 0.001, k=50, uncorrected; * refers to clusters survived at p < 0.05, FWE corrected; L=left, R=right, B=bilateral; brain regions are labeled according to the automated anatomic labeling toolbox for SPM8

| Brain Region | Hemisphere | Cluster Size | MNI Coordinates | | | BA | T-value |
| --- | --- | --- | --- | --- | --- | --- | --- |
|  |  |  | x | y | z |  |  |
| Inferior/Middle Frontal Gyrus | L | 84 | -34 | 24 | 22 | 45/46 | 4.62 |
| Middle Frontal Gyrus | L | 150 | -46 | 20 | 40 | 8/9 | 4.79 |
| Middle Frontal Gyrus/Frontal Pole | L | 79 | -38 | 54 | 6 | 10 | 4.34 |
| Superior/Middle Frontal Gyrus | L | 312 | -24 | 26 | 60 | 6/8/9 | 5.43* |
| Superior Frontal Gyrus | R | 112 | 20 | 66 | 10 | 10 | 5.60 |
| Inferior Parietal Lobule/Superior Parietal Lobule/Angular Gyrus/Supramarginal Gyrus | L | 620 | -32 | -74 | 50 | 7/39/40 | 5.47* |
| Middle Temporal Gyrus | R | 73 | 66 | -2 | -24 | 21 | 5.54 |
| Inferior Temporal Gyrus | R | 58 | 60 | -20 | -18 | 20/21 | 4.36 |
