# Supplementary material for: Helping or punishing strangers: neural correlates of altruistic decisions as third-party and of its relation to empathic concern
Source: Front Behav Neurosci. 2015 Feb 18;9:24. doi: 10.3389/fnbeh.2015.00024 (PMC4332347; doi:10.3389/fnbeh.2015.00024)
Supplement: Supplementary file 6 [file Table6.DOCX]

***Supplementary Material***

**Helping or punishing strangers: neural correlates of altruistic decisions as third-party and of its relation to empathic concern**

**Yang Hu^1*†^, Sabrina Strang^1,2 †^, Bernd Weber^1,3^**

^1^Center for Economics and Neuroscience, University of Bonn, Bonn, Germany

^2^Department of Psychology, University of Lübeck, Germany

^3^Department of Epileptology, University Hospital Bonn, Bonn, Germany

*** Correspondence:** Yang Hu, Center for Economics and Neuroscience, University of Bonn, Nachtigallenweg 86, Bonn, 53127, Germany.

[huyang@uni-bonn.de](mailto:huyang@uni-bonn.de)

^†^These authors are co-first authors.

1. **Supplementary Figures and Tables**

## Suplementary Tables

**Supplementary Table 6. Regions that enhanced functional connectivity with bilateral striatum during third-party help/punishment decisions.** Note: threshold is set to p < 0.001, k=50, uncorrected; * refers to clusters survived at p < 0.05, FWE corrected; L=left, R=right, B=bilateral; brain regions are labeled according to the automated anatomic labeling toolbox for SPM8.

| Seed Region | Brain Region | Hemisphere | | | Cluster Size | MNI Coordinates | | | BA | T-value |
| --- | --- | --- | --- | --- | --- | --- | --- | --- | --- | --- |
|  |  |  | | |  | x | y | z |  |  |
| Left Striatum | HELP > HELP_CONTROL | | | |  |  |  |  |  |  |
|  | Middle/Inferior Frontal Gyrus | | R | | 50 | 60 | 26 | 20 | 45/46 | 4.47 |
|  | Supplementary Motor Area | | B | | 78 | -8 | 4 | 64 | 6 | 4.71 |
|  | Middle/Posterior Cingulate Cortex | | B | | 319 | -8 | -26 | 46 | 24/31 | 4.73* |
|  | Superior Temporal Lobe/  Temporal Pole | | L | | 104 | -36 | 12 | -22 | 38 | 4.92 |
|  | Precuneus/Cuneus | | R | | 110 | 24 | -82 | 26 | 7/18/31 | 4.06 |
|  | Ligual Gyrus/ Fusiform Gyrus/ Cuneus | | B | | 3300 | 18 | -72 | -4 | 17/18/  19/37 | 6.79* |
|  | Thalamus | | B | | 81 | -4 | -4 | 10 |  | 5.27 |
|  |  | |  | |  |  |  |  |  |  |
|  | PUNISH > PUNISH_CONTROL | | | |  |  |  |  |  |  |
|  | Inferior Frontal Gyrus | | | L | 169 | -48 | 14 | 22 | 44/45 | 5.50* |
|  | Superior Frontal Gyrus | | | L | 76 | -22 | -4 | 52 | 6 | 4.45 |
|  | Precentral Gyrus | | | L | 528 | -44 | -4 | 44 | 6 | 6.44* |
|  | Precentral Gyrus | | | R | 270 | 40 | -4 | 40 | 6 | 5.80* |
|  | Superior/Middle  Temporal Gyrus | | | L | 323 | -58 | -34 | 2 | 21/22 | 5.33* |
|  | Middle Temporal Gyrus/  Temporal Pole | | | R | 105 | 58 | 10 | -18 | 21/38 | 6.63 |
|  | Ligual Gyrus/ Fusiform Gyrus/  Cuneus/Precuneus/  Parahimppocampa Gyrus | | | B | 12443 | 20 | -68 | -2 | 7/17/18/19/31 | 10.10* |
|  | Putamen/Amygdala | | | L | 101 | -18 | 8 | -6 |  | 4.54 |
|  | Putamen | | | R | 58 | 22 | 10 | -4 |  | 4.33 |
|  |  | | |  |  |  |  |  |  |  |
| Right Striatum | HELP > HELP_CONTROL | | | |  |  |  |  |  |  |
|  | Middle/Inferior Frontal Gyrus | | | R | 172 | 56 | 28 | 18 | 45/46 | 5.88 |
|  | Superior/Middle Frontal Gyrus | | | R | 71 | 28 | 40 | 42 | 8/9 | 4.77 |
|  | Supplementary Motor Area | | | L | 60 | -12 | 4 | 62 | 6 | 4.00 |
|  | Precentral Gyrus | | | R | 196 | 54 | -2 | 52 | 6 | 4.73* |
|  | Superior/Middle  Temporal Gyrus | | | L | 234 | -58 | -32 | 0 | 22 | 4.76* |
|  | Middle Teporal Gyrus/Temporal Pole | | | R | 76 | 48 | 4 | -18 | 21/38 | 4.94 |
|  | Inferior Temporal Gyrus/ Fusiform Gyrus | | | L | 277 | -36 | -36 | -20 | 20/36 | 5.18* |
|  | Parahippocampa Gyrus/  Fusiform Gyrus | | | R | 55 | 32 | -28 | -24 | 36 | 4.07 |
|  | Cuneus | | | R | 50 | 18 | -80 | 30 | 7/31 | 4.13 |
|  | Ligual Gyrus/ Fusiform Gyrus | | | B | 5026 | -6 | -74 | 2 | 17/18/  19/37 | 6.80* |
|  | Putamen/Insula/  Parahippocampa Gyrus | | | L | 352 | -28 | -20 | 0 |  | 6.23* |
|  | Caudate/Putamen | | | R | 57 | 22 | 14 | 8 |  | 4.70 |
|  |  | | |  |  |  |  |  |  |  |
|  | PUNISH > PUNISH_CONTROL | | | |  |  |  |  |  |  |
|  | Medial Orbital Frontal Gyrus/  Anterior Cingulate Cortex | | | B | 315 | 0 | 42 | -6 | 10/11/  32 | 6.37* |
|  | Precentral Gyrus | | | L | 278 | -40 | -6 | 38 | 6 | 4.73* |
|  | Precentral Gyrus | | | R | 135 | 34 | -2 | 34 | 6 | 6.07 |
|  | Middle Temporal Gyrus | | | R | 55 | 40 | -78 | 18 | 19 | 4.54 |
|  | Temporal Pole | | | L | 55 | -44 | 24 | -32 | 38 | 5.67 |
|  | Temporal Pole | | | R | 86 | 58 | 10 | -16 | 38 | 5.65 |
|  | Superior Parietal Lobule | | | L | 163 | -20 | -72 | 56 | 7 | 4.58 |
|  | Cuneus | | | R | 124 | 16 | -88 | 22 | 18 | 4.35 |
|  | Lingual Gyrus/ Fusiform Gyrus/  Precuneus/Cuneus/  Parahippocampa Gyrus | | | B | 4781 | -18 | -84 | 10 | 17/18/  19/23/  30/31 | 6.69* |
|  | Putamen | | | R | 59 | 26 | 10 | -4 |  | 4.64 |
