# Supplementary material for: Helping or punishing strangers: neural correlates of altruistic decisions as third-party and of its relation to empathic concern
Source: Front Behav Neurosci. 2015 Feb 18;9:24. doi: 10.3389/fnbeh.2015.00024 (PMC4332347; doi:10.3389/fnbeh.2015.00024)
Supplement: Supplementary file 7 [file Image1.PDF]

Supplementary Material

Helping or punishing strangers: neural correlates of altruistic decisions as third-party and of its relation to empathic concern

Yang Hu[1*†], Sabrina Strang[1,2 †], Bernd Weber[1,3]

[1]Center for Economics and Neuroscience, University of Bonn, Bonn, Germany

[2]Department of Psychology, University of Lübeck, Germany

[3]Department of Epileptology, University Hospital Bonn, Bonn, Germany

*** Correspondence:** Yang Hu, Center for Economics and Neuroscience, University of Bonn, Nachtigallenweg 86, Bonn, 53127, Germany.

huyang@uni-bonn.de

[†]These authors are co-first authors.

1. Supplementary Figures and Tables

1.1. Suplementary Figures

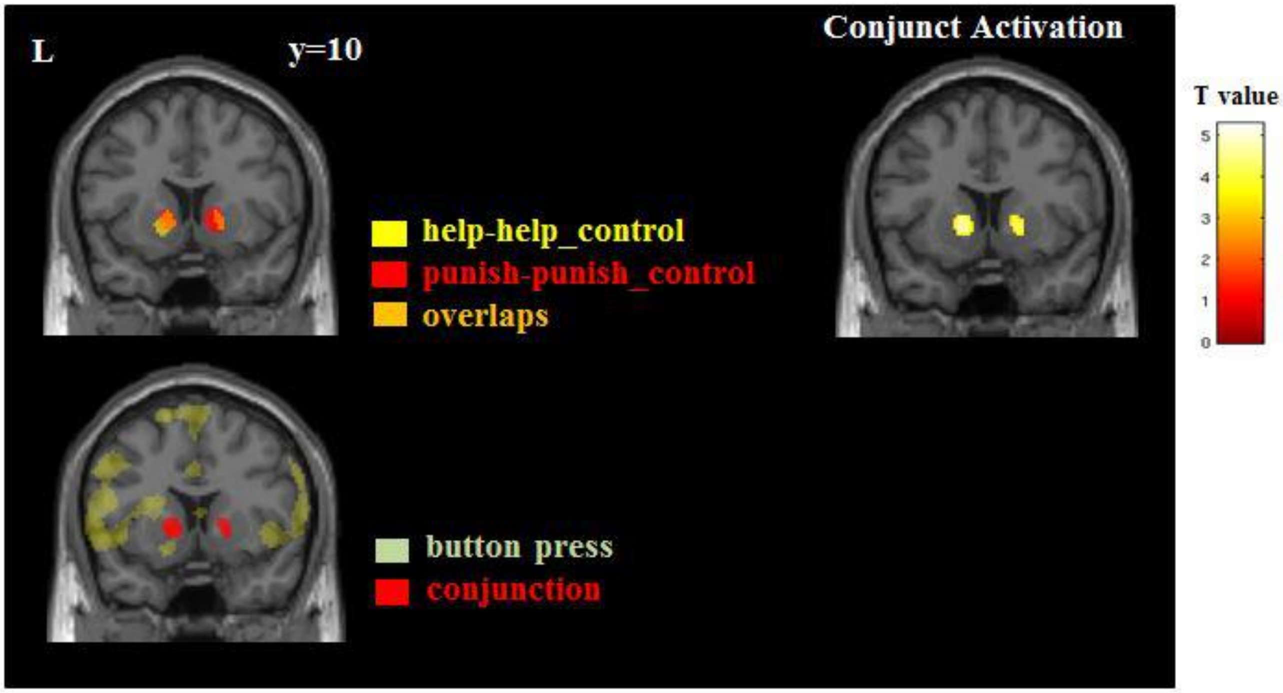

Supplementary Figure 1. Separate and conjunction mapping of bilateral striatum involved in third-party help and punishment after controlling the effect of button pressing during decision.
